# Evaluation of oil removal efficiency and enzymatic activity in some fungal strains for bioremediation of petroleum-polluted soils

**DOI:** 10.1186/1735-2746-9-26

**Published:** 2012-12-15

**Authors:** Fariba Mohsenzadeh, Abdolkarim Chehregani Rad, Mehrangiz Akbari

**Affiliations:** 1 Laboratory of Microbiology, Department of Biology, Bu-Ali Sina University, Hamedan, Iran; 2 Laboratory of Plant Cell Biology, Department of Biology, Bu-Ali Sina University, Hamedan, Iran; 3 Department of Biology, Islamic Azad University, Broujerd Branch, Broujerd, Iran

**Keywords:** Enzymatic activity, Fungi, Petroleum removing, Soil pollution

## Abstract

**Background:**

Petroleum pollution is a global disaster and there are several soil cleaning methods including bioremediation.

**Methods:**

In a field study, fugal strains were isolated from oil-contaminated sites of Arak refinery (Iran) and their growth ability was checked in potato dextrose agar (PDA) media containing 0-10% v/v crude oil, the activity of three enzymes (Catalase, Peroxidase and Phenol Oxidase) was evaluated in the fungal colonies and bioremediation ability of the fungi was checked in the experimental pots containing 3 kg sterilized soil and different concentrations of petroleum (0-10% w/w).

**Results:**

Four fungal strains, *Acromonium* sp., *Alternaria* sp., *Aspergillus terreus* and *Penicillium* sp., were selected as the most resistant ones. They were able to growth in the subjected concentrations and *Alternaria* sp. showed the highest growth ability in the petroleum containing media. The enzyme assay showed that the enzymatic activity was increased in the oil-contaminated media. Bioremediation results showed that the studied fungi were able to decrease petroleum pollution. The highest petroleum removing efficiency of *Aspergillus terreus*, *Penicillium* sp., *Alternaria* sp. and *Acromonium* sp. was evaluated in the 10%, 8%, 8% and 2% petroleum pollution respectively.

**Conclusions:**

Fungi are important microorganisms in decreasing of petroleum pollution. They have bioremediation potency that is related to their enzymatic activities.

## Background

Petroleum pollution is a global disaster that is a common phenomenon in the oil-bearing and industrial regions [1]. Petroleum pollution of environments is dangerous for plants, animals and people [2,3]. Iran, as an oiled country, contained a lot of petroleum polluted-environments and the pollutions are increasing in recent years [4].

There are several soil cleaning methods including burning, washing, chemical applying and bioremediation [5]. Bioremediation is using of plants and microorganisms to remove or detoxify environmental contaminants. Bioremediation has been intensively studied over the past two decades, driven by the need for a low-cost, sustainable with natural environment, and *in-situ* alternative to more expensive engineering-based remediation technologies [1,6,7]. Bioremediation has been applied to remove crude oil [8-11], motor oil [12], and diesel fuel [13] from soil but the removal efficiency is highly variable [14].

Bioremediation of petroleum-polluted media were done using plants or plant-associated micro flora [15,16]. There are different economically and environmentally important uses for microorganisms, such as remediation and rehabilitation of petroleum contaminated soils [11,17-22].

Bioremediation of petroleum-contaminated soils is mainly based on biodegradation by the fungal strains that are present in the associated with plants or in the soils of petroleum polluted sites [23]. Some prior researchers reported that some fungal species are resistant to petroleum-pollution and they are capable to remove soil pollution. The results of Ulfig et al. [24] indicated that keratinolytic fungi, especially *Trichophyton ajelloi,* is a potential tool for assessment of soil petroleum hydrocarbon contamination and associated bioremediation progress. Fungal strains namely *Alternaria alternate*, *Aspergillus flavus*, *Curvularia lunata*, *Fusarium solani*, *Mucor racemosum*, *Penicillium notatum* and *Ulocladium atrum* were isolated from the soils in the petroleum polluted areas in Saudi Arabi [25]. Eggen and Majcherczykb [17] showed that white rot fungus, *Pleurotus ostreatus,* was able to remove polycyclic aromatic hydrocarbons (PAH) from contaminated soil. Little attention has been paid to the role of fungal species in the environmental biotechnology and bioremediation of petroleum pollution, specially in Middle Eastern region [18,25]. Some fungal strains including *Alternaria alternate*, *Aspergillus flavus*, *Curvularia lunata*, *Fusarium solani*, *Mucor racemosum*, *Penicillium notatum* and *Ulocladium atrum* were isolated from the soils in the petroleum-polluted areas in Iran [11]. The aim of this research was to collect fungal strains from petroleum-polluted soils of Arak refinery, evaluation of their ability in removing of petroleum pollution in experimental conditions and determination of their enzymatic activity during petroleum removing.

## Methods

### The studied area

The Arak oil refinery, located near the Arak city in the center of Iran was selected in this study. The city is located in the central part of Iran (34° 5' 8" North, 49° 41' 2" East) with elevation average about 1723 meters above sea level. The population of the city is 503673. Arak is the capital city of Markazi province and is mostly arid or semiarid, subtropical along Caspian coast. It rains most in winter and is moderately warm in summer. Its annual precipitation is 317.7 mm, mean annual temperature is 11.8°C and 46% humidity.

Arak oil refinery is located at 25 km far away from Arak city. Arak refinery is a relative new refinery with the production capacity of 22434 barrel in day that funded in 1992. Soil characters of the area was evaluated as sandy loam containing 80% sand, 12% loam, 6% sludge and 2% organic material with pH 6.8. Chemical composition of the used crude oil in the refinery is 13.4% saturated hydrocarbons, 40% aromatic hydrocarbons, 46.6% polar compounds (Refinery office data). Due the oil refining activities in this region, a high degree of petroleum pollution (5-10%) was reported in the refinery areas [16]. The identification of soil contamination was also possible based on a visual examination of the soil.

### Selection of fungal strains

Since the amounts of microorganisms in the around of plant roots are up to 200 times more than soil [13], root samples were harvested from the plants growing in the polluted area of Arak refinery, and sliced into segments with 1 cm length, washed and then dried. The samples were kept in Sodium hypo chloride 1% (30 sec) and then ethanol 70% (30 sec), for removing the peripherally attached microorganisms, and dried after washing with distilled water [13]. The samples were kept in potato dextrose agar (PDA) media containing lactic acid. The Petri dishes were incubated in 25 ± 2°C for 4 days. Then, different fungal colony were isolated and cultured separately in PDA [16]. Fungal specimens were examined under light microscope after preparations and identified using morphological characters and taxonomical keys provided in the mycological keys [26-28]. The specimens were also sent to the department of mycology in our university for confirmation of their scientific names.

### Determination of the fungal growth ability under petroleum pollution

The growth assay was used to find the resistant fungal species to petroleum contamination of the soil. The assays were conducted by comparing the growth rates of fungal strains, as colony diameter, on the oil contaminated and control Petri dishes. Test dishes were prepared by adding crude oil to warm PDA solution. In order to have a uniform concentration of oil in all plates, the solution was thoroughly mixed with a magnetic stirrer, right before it was added to the plates. Different concentrations of oil/PDA mixture (2, 4, 6, 8 and 10% v/v) were prepared. Pure PDA was used in control plates. All dishes were incubated with 2 mm plugs of fungal mycelia taken from agar inoculums plate. The dishes were incubated at 25±2°C in an incubator. Fungal mycelia extension on the plates (colony diameter) was measured using with measuring tape after 7 days and compared with the control plates.

### Evaluation of petroleum removing

The four fungal strains that showed the highest resistant and growth ability in the prior stage, were chosen for this study. They are common and native fungi that isolated from the studied petroleum polluted area. Ninety-six pots were selected for this study and divided in to four groups; each group containing 24 pots and used for each fungal strain. Each pot was filled with 3kg of sterile agricultural soil and mixed with 3g of the studied fungi. The experimental groups were as groups A, B, C, and D. Each group including one of the above-mentioned fungal strains and sub-groups are growing in the pots added different concentrations (0, 2, 4, 6, 8 and 10% w/w) of crude oil.

The pots were incubated in a greenhouse in the temperature of 25±2°C for three months. The soil of experimental and control pots were homogenized separately and were kept in 4°C at refrigerator until future study. Concentrations of crude oil (TOG%) were determined and compared in the soil of experimental and control pots.

### Determination of Total Oil and Grease (TOG)

The soil samples from experimental and control pots were collected separately. Each sample, without fungal segments, was homogenized and stored at 4°C until further processing. TOG was analyzed according to the EPA method 9071 A and EPA Method 3540 B [29]. Five gram of the soils in two replicates were acidified with hydrochloric acid to pH=2 and dehydrated with magnesium sulphate monohydrate. After 15 min, samples were transferred into paper extraction thimbles and placed into a Soxhlet type apparatus. TOG was extracted with dichloromethane for 8 h. The extract was filtered through filter paper (Whatman No. 4) with 1g sodium sulphate. The solvent was evaporated with a rotary evaporator and the weight of dry extract was determined. Percentage of TOG decreasing was calculated based on soil weight and compared in the experimental and control pots.

### Determination of enzymatic activity

For the assays of Catalase (CAT) and Peroxidase (POX) enzymes, mycelia (200 mg) was homogenized in an ice-cooled mortar, grounded in 1 ml of 100 mM potassium phosphate buffer (pH 7.4) and centrifuged at 10,000 rpm for 10 min under cooling and the supernatant was used for enzyme assay. The activities of CAT and POX were determined according to Aebi [30]. CAT activity was determined by measuring the decomposition of H_2_O_2_ and the decline in absorbance at 240 nm was followed for 3 min. The reaction mixture contained 50 mM phosphate buffer (pH 7.0), 15 mM H_2_O_2_, and 0.1 ml of enzyme extract, was used which started the reaction in 3 ml. The activity of POX evaluated by measuring the oxidation of guaiacol and the increase in absorbance at 470 nm was recorded for 3 min. The reaction mixture contained 50 μl of 20 mM guaiacol, 2.8 ml of 10 mM phosphate buffer (pH 7.0), and 0.1 ml enzyme extract. The reaction was started with 20 μl of 40 mM H_2_O_2_. The activity was defined as differences of optical density per min, for each mg of fresh weight of samples (Δ OD/ min/ mg FW).

The Phenol Oxidase (POD) activity was studied in the extracts of fungi growing in PDA media with different concentrations. The procedure adopted by Tate [31] and Theorell [32] was followed. Pure standard horseradish POD (SIGMA, USA) of RZ value 3.04 was used as standard. The POD activity was measured using guaiacol (1.11 mg/ml density) as chromogenic on spectrophotometer. The extract free of all cellular components was heated at 65°C for three minutes in a water bath and then cooled promptly by placing in ice bucket for inactivation of catalase activity.

### Statistical analysis

In order to detect a significant difference between the experimental groups and control ones analysis of variance (ANOVA) followed by the least significant difference test (LSD) that was performed between studied groups [33]. Each data was represented as the means ± SD of 5 samples for experimental groups and also 5 for control.

## Results

### Isolated fungi

The fungi growing in the petroleum-polluted areas of Arak refinery were isolated and their growth ability was checked under petroleum pollution. Four fungal strains that showed the highest abundance in the polluted area and also the highest growth ability were chosen and identified by morphological characters and taxonomical keys. The results of the taxonomic determination for the fungi showed that the selected fungal species that present in the petroleum polluted soils are: *Acromonium* sp.*, Alternaria* sp.*, Aspergillus terreus* and *Penicillium* sp.

### Fungal growth ability under petroleum pollution

**Figure 1:**
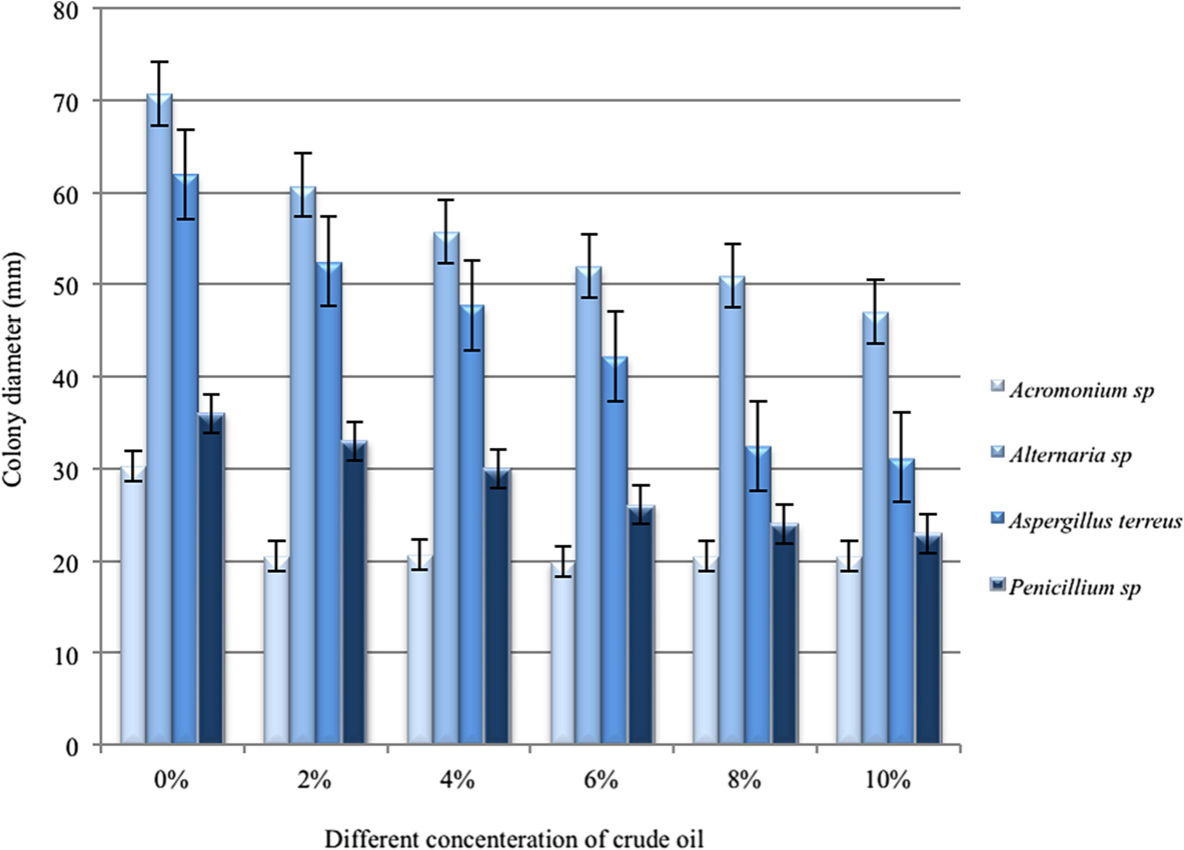
**The growth ability of the isolated fungal strains under different percentage of petroleum pollution.** Results showed that *Alternaria* sp. has the highest and *Acromonium* sp. the lowest growth ability. Each data represented the mean±SE of five samples.

The growth ability of the isolated fungal strains was carried out under different concentrations of crude oil and was expressed as diameter of the colony (Figure 1). The results showed that the all above-mentioned fungi are resistant to petroleum pollution and they made a sufficient colony in 2% crude oil concentration; meanwhile, only some of them are resistant to higher pollution. Among the studied fungi, *Alternaria* sp. showed the highest resistance to 10% petroleum pollution (with 47.50 mm diameter of colony after 7 days growth), and three fungal strains including *Aspergilus terreus* (31.25 mm), *Penicilium* sp. (23 mm), and *Acromonium* sp. (20.50 mm) were also relatively resistant ones. The colony diameters were determined after 7 days growth in the different concentrations of petroleum polluted PDA media (Figure 1).

### Bioremediation

**Figure 2:**
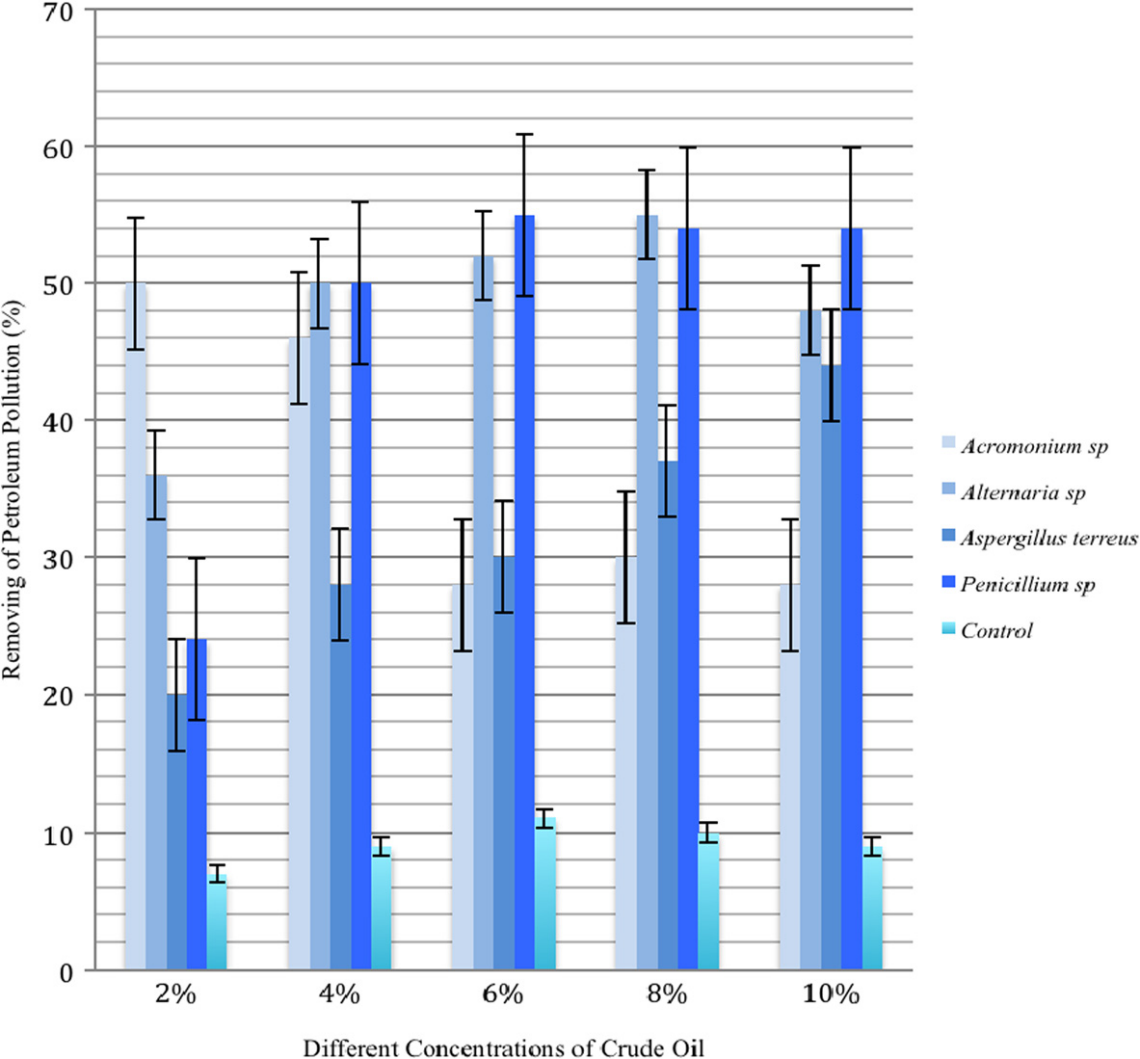
**Petroleum removing (%) by the studied fungal strains.** Results showed that *Penicillium* sp. and *Alternaria* sp. are more effective fungai in high petroleum-polluted media. Each data represented the mean±SE of five samples.

Three months after growing of fungal strains in petroleum-contained soils, concentration of petroleum was determined in the experimental pots and compared with the beginning of experiment. The obtained data showed that the concentration of petroleum was decreased considerably in the all experimental pots (Figure 2). The data showed that the all studied fungal strains were able to decrease petroleum pollution. For *Aspergillus terreus*, the most decreasing of petroleum was evaluated in the pots containing 10% petroleum (44% decreasing) and the lowest decrease was in the pots containing 2% petroleum (20%) (Figure 2). *Acromonium* sp. was also cause to decrease amount of crude oil in the growing pots. The highest removal ability was in the pots containing 2% petroleum (50% decreasing) and the lowest one was in the pots with 10% pollution (28%) (Figure 2).

For *Alternaria* sp. petroleum removing in the pots with 8% crude oil is the highest (55% decreasing) and the lowest decrease (50%) was in the pots with 2%. Finally, for *Penicillium* sp., the highest decreasing of petroleum was evaluated in the pots containing 8% petroleum (54% decreasing) and the lowest decrease was in the pots containing 2% petroleum (25%) (Figure 2). Based on the results, the all fungi are effective in petroleum removing from soil of the pots.

### Enzymatic assay

The activity of three enzymes, catalase (CAT), peroxidase (POX) and polyphenol oxidase (POD), were determined in the above-mentioned fungi during the growth in the media with different concentrations of petroleum pollution. After two weeks growing in the media containing crude oil, enzymatic activity were determined in the colonies of experimental Petri dishes and compared with control ones. Results showed that the activity of the enzymes was increased considerably in the all-experimental Petri dishes (Table 1). For *Acromonium* sp., the highest activity of catalase (CAT) was in the group growing in 2% petroleum pollution and it was decreased in the other experimental groups armed with increasing of crude oil concentrations. The lowest activity was observed in the control group. In *Alternaria* sp. activity of CAT was increased with increasing of petroleum pollution. The highest activity was determined in the colonies growing in the media containing 8% crude oil, and in the all-experimental groups were more than control ones (Table 1). Similar pattern was also observed for *Penicillium* sp. and its highest CAT activity was in the group treated with 8% crude oil. In *Aspergillus terreus*, straight relationship was observed between the CAT activity and crude oil concentrations (Table 1). The lowest activity was observed in the control group and the highest was in the group treated with 10% petroleum pollution.

**Table 1:** Different Enzyme activity (Unit/mg) in fungal strains under different petroleum concentrations

	**0%**			**2%**			**4%**			**6%**			**8%**			**10%**		
	**A**	**B**	**C**	**A**	**B**	**C**	**A**	**B**	**C**	**A**	**B**	**C**	**A**	**B**	**C**	**A**	**B**	**C**
*Acromonium* sp.	2±0.1	13±2	24±3	7.5±0.3	28±2	13±3	6±1.5	26±3	12±2	5±0.3	13±2	7±1	3.2±0.2	12±1	6±1	3±o.2	11±2	5±1
*Alternaria* sp.	1.3±0.2	11±2	3±0.2	2.4±0.4	22±3	8±1.4	2.5±0.4	24±3	9±1	4±0.5	28±2	11±0.8	6.2±0.2	40±2	15±2	5±0.2	32±3	12±2
*Aspergillus terreus*	1.5±2	25±0.3	6±1	**30±2**	33±3	11±2	3.8±0.3	35±2	13±3	4±0.3	36±4	16±2	4.4±0.2	36±4	18±2	6.5±0.2	40±3	24±5
*Penicillium* sp.	3±0.1	32±1	12.5±2	3.2±0.2	51±5	14±2	4±0.2	68±4	16±1	4.1±0.2	70±3	24±2	8.5±0.1	88±5	35±4	4.5±0.2	58±4	24±3

A, Catalase; B, Phenol Oxidase; C, Peroxidase.

Results showed that the activity of peroxidase (POX) in the fungal strains growing in the petroleum-polluted media, was different with control ones. In *Acromonium* sp. (Table 1), POX activity was decreased with increasing of petroleum pollution and the highest activity was in non-polluted media. In *Alternaria* sp., POX activity was increased with increasing of petroleum pollution. The highest activity was observed in the group with 8% pollution but then decreased in the group containing 10% pollution. The lowest activity was evaluated in control group. In *Aspergillus terreus,* POX activity was increased with petroleum pollution straightly (Table 1). So, the highest activity was in 10% pollution and the lowest one was in non-polluted group. In *Penicillium* sp. the highest activity was in the group containing 8% petroleum pollution and the activity of POX in the groups with 6 and 10% was also higher than non-polluted group but in the groups with 2 and 4% pollution are near to control ones (Table 1).

Phenol oxidase (POD) activity was compared in the fungi growing in petroleum-polluted and control media. Results showed that in *Acromonium* sp. the highest activity was in the groups treated whit 2 and 4% petroleum. In other experimental groups its activity was similar with control ones (Table 1). In *Alternaria* sp., POD activity was increased with increasing the petroleum pollution until 8%, but it was decreased slightly in the group growing in media with 10% petroleum pollution (Table 1). In *Aspergilus terreus*, POD activity was increased with increasing of petroleum pollution and the highest activity was determined in the group growing in media with 10% petroleum pollution. Finally, for *Penicillium* sp., the highest POD activity was evaluated in the group growing in the media containing 8% petroleum pollution and the lowest activity was in the group growing in the non-polluted media (Table 1).

## Discussion

Study on the fungal species showed that *Acromonium* sp., *Alternaria* sp.*, Aspergillus terreus* and *Penicillium* sp. were the common fungi, with high frequency in the petroleum polluted areas. It seems that petroleum pollution could not inhibit the growth and variation of fungal strains in petroleum polluted areas. It seems that the fungal species used oil compounds as nutrients and petroleum pollution cause to increase fungal growth. The similar results were reported by some researchers [11,16-21]. *Penicillium oxalicum* was also isolated from petroleum-polluted soils and reported as degradability potential microorganism for bioremediation of crude oil [34].

The *In vitro* growth test of the isolated fungi showed a species-specific response. All of the studied fungal strains were able to growth in 2% v/v oil pollution and therefore could be useful for the remediation of light soil pollution. Although the growth of fungal species were reduced by increasing oil concentrations (more than 4% v/v), but all of them were still able to growth in the high concentrations of petroleum. They were produced sufficient colonies in the high-polluted media but with a lagging time. It seems that they could be used also for oil degradation in the soils with high pollution effectively. Our results are accordance with the some finding of other researchers about other different fungal species [11,16-21].

Results of this research showed that the amounts of petroleum pollution were decreased in the presence of the studied fungal strains considerably. It means that the fungal strains were able to degrade crude oil and consumption of its components. Although there are several reports about the fungal ability in removing of petroleum and its derivers from the polluted soils [11,16,18,20,21], but this is the first report about the petroleum removing ability of the studied fungal strains. The results of our study proposed the above-mentioned fungi for using in remediation of petroleum-polluted environments in a field study. It means that the data of this study indicated that isolated fungi *Acromonium* sp., *Alternaria* sp.*, Aspergillus terreus* and *Penicillium* sp. may have the potential for bioremediation of soil in highly polluted conditions especially in semi-dry regions.

Enzymatic assay indicated that activities of the all studied enzymes were increased with the increasing of petroleum pollution. In *Acromonium* sp., only the activity of catalase was decreased with the increasing of petroleum pollution. In other fungal strains, there is a sharp rise of enzymatic activity in the petroleum-polluted media. *Penicillium* sp. showed the highest catalase activity and the lowest one is observed in *Aspergillus* sp. For peroxidase and phenol oxidase, the highest activity was evaluated in *Penicillium* sp. and the lowest one was in *Acromonium* sp. Kotik et al. [35] reported that enzymatic activity of microorganisms were increased in the petroleum polluted soils and they were able to find hydrolase epoxide as a new enzyme that has major role in the degradation of crude oil. High activity of catalase and peroxidase was also reported in the soil microorganisms in petroleum-polluted soils [36,37] that is accordance with our results. Tang et al. [38] applied ryegrass and effective microorganisms for bioremediation of petroleum polluted soils and increasing of enzymatic activity was observed in the soil microorganisms including *Edwardsiella tarda*, *Bacterium aliphaticum*, *Bacillus megaterium*, *Bacillus cereus*, *Pseudomonas maltiphilia*, *Fusarium vertiaculloide*, *Botryodiphodia thiobroma*, *Fusiarum oxysporum*, *Cryptococcus neofomas*, *Aspergillus niger* and *Candida tropicalis.* Ugochukwu et al. [36] reported that biochemical analysis revealed that except *B. aliphaticum,* which had high lipase activity, fungal isolates generally recorded higher lipase activities than bacterial isolates.

Based on the results of this study, it seems that fungal strains are the most effective organisms that are abundant in petroleum polluted soils and they were able to digest and remove petroleum compounds enzymatically. Based on our results they were able to grow in petroleum-polluted media effectively and *Alternaria* sp. is the most resistant fungal strain to high degree of petroleum pollution than others. Bioremediation tests with the fungal strains showed that they are effective in decreasing of petroleum pollution from environment and based on the results *Alternaria* sp. and *Penicillium* sp. were the most effective ones. This means that the fungal strains had bioremediation potency for petroleum-polluted media and their enzymatic activity have a major role in degradation of petroleum.

## Conclusion

Our results showed that the studied fungi were able to growth in the subjected petroleum concentrations and *Alternaria* sp. showed the highest growth ability in the petroleum containing media. Results of bioremediation tests showed that the studied fungi were able to decrease petroleum pollution. The highest petroleum removing efficiency of *Aspergillus terreus*, *Penicillium* sp., *Alternaria* sp. and *Acromonium* sp. was evaluated in the 10%, 8%, 8% and 2% petroleum pollution respectively. Enzymatic activities were increased in the fungal colonies growing in the oil-polluted media; this means that the fungal enzymes have a critical role for petroleum degradation.

## Competing interests

The authors declare that they have no competing interests.

## Authors’ contributions

MA, is a MSc student and this manuscript was wrote based on her thesis results. She analyzed the Samples. ACR, is the supervisor of the thesis and supervised the methods and the project. He wrote the original research plan of the project and also edited the manuscript. FM, is co-supervisor of the thesis. She wrote the original manuscript and participated in planning of sample analysis. All authors read and approved the final manuscript.
